# Enhancing immune protection in dairy cattle: role of *Escherichia coli* Nissle 1917 in boosting the efficacy of a *Mycoplasma bovis*-BoAHV-1 combined vaccine

**DOI:** 10.1128/msphere.00178-26

**Published:** 2026-04-24

**Authors:** Sen Zhang, Guoxing Liu, Jianguo Chen, Aizhen Guo, Yingyu Chen

**Affiliations:** 1 National Key Laboratory of Agricultural Microbiology, Hubei Hongshan Laboratory, College of Veterinary Medicine, Huazhong Agricultural University 627716, Wuhan, China; 2 Hubei International Scientific and Technological Cooperation Base of Veterinary Epi Demiology, Cooperative Innovation Center for Sustainable Pig Production, Wuhan, China; 3 Key Laboratory of Development of Veterinary Diagnostic Products, Ministry of Agriculture and Rural Affair, Wuhan, China; University of Wyoming College of Agriculture Life Sciences and Natural Resources, Laramie, Wyoming, USA

**Keywords:** bovine respiratory disease, *Escherichia coli *Nissle 1917, combined vaccine, immunogenicity, protective efficacy

## Abstract

Bovine respiratory disease (BRD) ranks among the most prevalent and economically burdensome diseases affecting the global dairy cattle industry, primarily resulting from co-infection with multiple pathogens, including *Mycoplasma bovis* (*M. bovis*) and bovine alphaherpesvirus type 1 (BoAHV-1). Despite the availability of vaccines, promoting their immunogenicity to deliver rapid, effective protection remains a critical challenge. The non-pathogenic probiotic *Escherichia coli* Nissle 1917 (EcN) has exhibited immunomodulatory properties related to digestive health; however, its potential as an adjuvant in bovine respiratory applications remains unexplored. In this study, we investigated the role of EcN as an immune enhancer in dairy calves for the first time, emphasizing its effects on both innate and adaptive immune responses when administered alongside an *M. bovis*-BoAHV-1 vaccine. Our findings revealed that all calves maintained normal clinical signs following EcN administration. Compared to the blank control, EcN treatment significantly upregulated natural immune markers. Furthermore, the combination of EcN with the *M. bovis*-BoAHV-1 vaccine elicited superior adaptive responses, evident in the boosted intensity and duration of both cellular and humoral immunity. Under experimental challenge conditions, the EcN-treated vaccinated group showed a more robust specific immune response, along with lower pathogen shedding titers and shorter duration compared to calves receiving the combined vaccine alone. In conclusion, EcN can safely and effectively activate the innate immune response of dairy calves, improving the adaptive immune response induced by a combined vaccine. Our findings represent the first confirmation of EcN’s applicability in BRD and offer a novel strategy for enhancing vaccine protective efficacy.

**IMPORTANCE:**

Bovine respiratory disease is a major cause of economic losses in the global cattle industry, partly because of the delayed efficacy of current vaccines. This study investigated the intranasal administration of the probiotic *Escherichia coli* Nissle 1917 to enhance bovine immunity. The results illustrated that this probiotic safely activated the innate immune system, bridging the gap in protection between vaccination and the development of specific immune responses. When paired with a combined vaccine targeting *Mycoplasma bovis* and bovine alphaherpesvirus type 1, the probiotic significantly elevated protective antibody levels and reduced pathogen shedding and duration. These findings introduce an innovative non-antibiotic strategy for enhancing respiratory health in the cattle industry, providing a valuable tool to improve disease resistance and potentially reduce reliance on antimicrobial drugs.

## INTRODUCTION

Bovine respiratory disease (BRD) poses a significant threat to cattle health and the dairy cattle industry, leading to considerable morbidity and mortality (1). In China, the primary pathogens implicated in BRD are *Mycoplasma bovis* (*M. bovis*) and bovine alphaherpesvirus type 1 (BoAHV-1). *M. bovis* was first isolated from affected cattle in 2008 and has become widespread, with incidence rates reaching as high as 50% to 100% in certain regions (2, 3). A serological survey of BoAHV-1 revealed an overall prevalence of 40% across China (4), while in northeast China, this virus constitutes 45.2% of BRD-associated viruses identified in lung samples from diseased cattle (5). Our previous studies developed an attenuated and marker combined live vaccine targeting *M. bovis* and BoAHV-1, which displayed promising protective efficacy in dairy calves (6). Nevertheless, enhancing the immune response continues to present a tough challenge.

Current vaccines typically require multiple doses and may take time to confer adequate immunity, underscoring the necessity for strategies that facilitate immediate and robust immune responses. *Escherichia coli* Nissle 1917 (EcN), a nonpathogenic probiotic, has emerged as a promising enhancer of immunogenicity in vaccine applications. It plays a vital role in regulating the host immune response by balancing the secretion of immune factors and improving overall immune capacity, thereby aiding in the management of inflammation (7, 8). Moreover, EcN’s ability to stimulate and activate both innate and adaptive immune responses renders it a valuable asset in combating infections (9). Its distinctive properties and capability to modulate chemokine expression (10) suggest that EcN can markedly bolster host immune defenses when utilized as a vaccine adjuvant.

More importantly, the therapeutic effects of EcN extend beyond the digestive system; intranasal administration has been shown to effectively activate the innate immune response in the lungs, holding promising potential for prompt protection against respiratory pathogens (11, 12). This characteristic positions EcN as a potentially effective means of delivering antigens that can stimulate strong mucosal immunity.

In this study, we explored the innovative application of EcN as an adjuvant in a combined vaccine targeting *M. bovis* and BoAHV-1 for the first time, aiming to assess whether the co-administration of EcN with the combined vaccine could safely stimulate the host innate immune system while enhancing the adaptive immune response necessary for robust protection, thereby addressing the critical limitation of delayed immune responses observed in current vaccination protocols. Ultimately, these findings seek to establish an innovative non-antibiotic vaccine immunization strategy that bridges the gap between vaccination and effective immunity, offering a solution to mitigate the devastating impact of BRD.

## MATERIALS AND METHODS

### Cells, viruses, and vaccines

The wild-type *M. bovis* strain HB0801 (GenBank accession number: CP002058.1), the *M. bovis* strain HB150, the wild-type BoAHV-1 strain HB06 (GenBank accession number: AJ004801.1), and the BoAHV-1 strain gG-/tk- were maintained at the National Key Laboratory of Agricultural Microbiology. Madin‒Darby bovine kidney (MDBK) cells were obtained from the China Institute of Veterinary Drug Control.

The *M. bovis*-BoAHV-1 combined vaccine was stored at the National Key Laboratory of Agricultural Microbiology and prepared as previously described (6).

### Culture of *M. bovis*, BoAHV-1, and EcN

*M. bovis*, BoAHV-1, and EcN were cultured as previously described (13–15). Briefly, the wild-type *M. bovis* HB0801 and the *M. bovis* HB150 strains were cultured in PPLO complete medium at 37°C in a 5% CO_2_ incubator for 40–48 h. BoAHV-1 strains HB06 and BoAHV-1 gG-/tk- were cultured in Dulbecco’s modified Eagle’s medium supplemented with 10% fetal bovine serum in MDBK cells at 37°C in a 5% CO_2_ incubator. EcN was cultured in Luria–Bertani medium at 37°C under the same CO_2_ conditions.

### Animal experiments

A total of 27 Holstein dairy calves (13 females and 14 males), aged 2 to 4 months, and seronegative for *M. bovis* and BoAHV-1, were randomly assigned to nine groups. To prevent cross-infection, all the calves were housed in isolation. The detailed study design is summarized in Table 1.

**TABLE 1:** Animal immunization and challenge information

Group	No.	Vaccination content	Challenge strain and dose
1	3 (2 male and 1 female)	EcN + combined vaccine	1.0 × 10^9^ CFU *M*. *bovis* HB0801
2	3 (1 male and 2 female)	EcN + combined vaccine	4.0 × 10^7^ TCID_50_ BoAHV-1 HB06
3	3 (2 male and 1 female)	Combined vaccine	1.0 × 10^9^ CFU *M*. *bovis* HB0801
4	3 (1 male and 2 female)	Combined vaccine	4.0 × 10^7^ TCID_50_ BoAHV-1 HB06
5	3 (2 male and 1 female)	EcN	1.0 × 10^9^ CFU *M*. *bovis* HB0801
6	3 (1 male and 2 female)	EcN	4.0 × 10^7^ TCID_50_ BoAHV-1 HB06
7	3 (2 male and 1 female)	PBS	1.0 × 10^9^ CFU *M*. *bovis* HB0801
8	3 (1 male and 2 female)	PBS	4.0 × 10^7^ TCID_50_ BoAHV-1 HB06
9	3 (2 male and 1 female)	PBS	\^*a*^

^*a*^ "\" indicates that the challenge of *M. bovis* HB0801 or BoAHV-1 HB06 is not applied.

Based on the established dosage from and with appropriate adjustments (11), calves in the EcN + combined vaccine and EcN groups were administered 2 mL of EcN (1 × 10^9^ CFU) intranasally via a 2 mL syringe on a single day; each nasal cavity was inoculated with 1 mL of EcN.

The combined vaccine was prepared in a 2 mL saline solution, with 1 mL administered into each nasal cavity of cattle in the EcN + combined vaccine and combined vaccine groups via a 2 mL syringe. Vaccination for the EcN + combined vaccine group took place the day following the completion of EcN inoculation. The non-immune and blank control groups received 2 mL of PBS via the same procedure.

**Fig 1:**
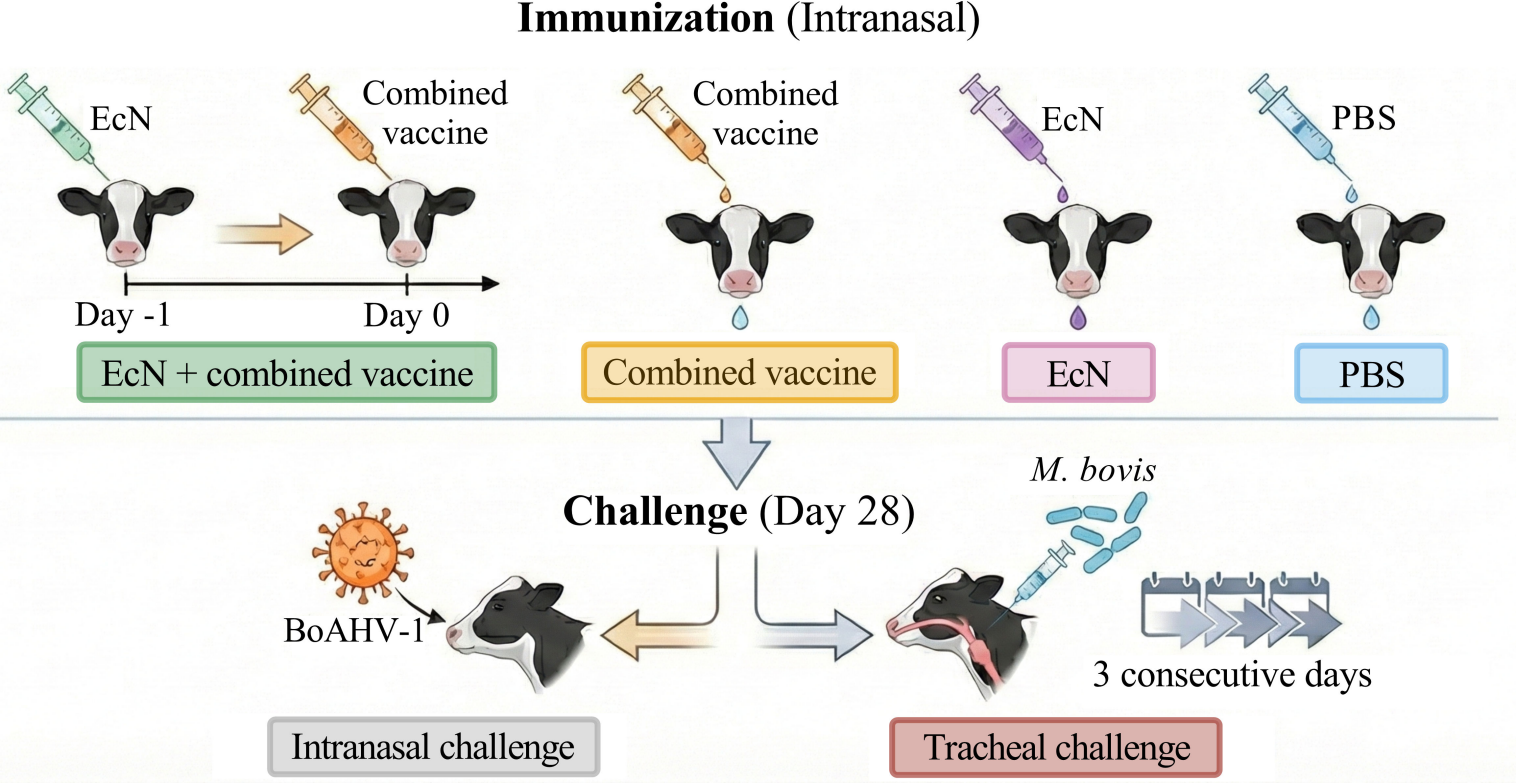
A schematic diagram illustrates the dairy calves receiving EcN and the combined vaccine, as well as undergoing challenge.

Groups 2, 4, 6, and 8 received a challenge of 4.0 × 10^7^ TCID_50_ BoHV-1 HB06 strain intranasally at 28 days after immunization, and tracheal injection challenge was performed on groups 1, 3, 5, and 7 with 1.0 × 10^9^ CFU *M*. *bovis* HB0801 strain at 28 days post-vaccination over three consecutive days. A schematic diagram of animal immunization and challenge is shown in Fig. 1.

### Clinical evaluation and sample collection

Clinical signs, including rectal temperature, respiratory status, and mental alertness, were continuously monitored throughout the experimental period. Rectal temperature was measured by inserting a thermometer approximately 10 cm into the rectum until the reading stabilized. Measurements were taken in the morning and afternoon before feeding, and the average temperature was recorded. Besides, mental alertness was qualitatively assessed daily based on the calves’ posture and responsiveness to external stimuli. The assessment was categorized into three levels: (i) normal/alert: bright, alert, and responsive to visual and auditory stimuli (e.g., human approach), with normal head position and active interaction with the environment; (ii) mildly depressed: quiet but responsive, exhibiting slightly droopy head or slower reactions to stimuli; (iii) severely depressed: dull appearance, head down, isolated from the group, reluctant to stand or move, and showing delayed or no response to external stimuli.

Nasal lavage fluid (NALF) was collected at baseline (day 0) and at regular intervals (days 7, 14, 21, and 28) with the established procedures following inoculation (16). One person held the calf’s head while another injected 5 mL of PBS containing 0.1% BSA into each nostril. The nasal cavity was gently cleaned for approximately 15 s, after which the calf’s head was lowered, and the effluent was collected. The process was repeated three times to obtain sufficient NALF (3–4 mL).

Nasal swabs were collected daily in the morning after feeding over a 28-day period following challenge.

Procoagulant blood samples were collected weekly until the end of the experiment. These samples were used to detect antibodies. Additionally, procoagulant blood samples for cytokine detection were collected on days 1 and 3 post-immunization; anticoagulated samples containing sodium heparin and samples containing EDTA were taken on days 7 and 14 post-immunization for the analysis of lymphocyte proliferation and lysozyme activity, respectively.

### Routine blood examination

The EDTA anticoagulated blood samples collected weekly after vaccination were analyzed via an automatic five-class blood cell analyzer (Mindray Biomedical Electronics Co., Ltd., Shenzhen, China) for routine blood examinations.

### Lymphocyte proliferation assay

Peripheral blood mononuclear cells (PBMCs) were isolated from sodium heparin anticoagulated blood samples. The blood was centrifuged at room temperature for 10 min to separate the components, and the middle cell layer was collected. To lyse erythrocytes, 4–6 times the volume of commercial erythrocyte lysate (Dakewe biotech Co., Ltd., Shenzhen, China) was added to the collected cell layer, mixed thoroughly, and incubated at 4°C for 2 min. Then, the mixture was centrifuged at 4°C for 10 min to collect PBMCs, and the supernatant was discarded. This process was repeated until complete lysis of erythrocytes occurred. The supernatant was discarded, and the PBMCs were washed twice with 10 mL of 1640 complete medium.

Isolated PBMCs (1.0 × 10^6^ cells) were cultured in each well of a 96-well cell culture plate. Each well received 10 μL of medium containing either concanavalin A (25 μg/mL), *M. bovis* whole-cell protein (25 μg/mL), or ultraviolet-irradiated inactivated BoAHV-1 (1.0 × 10^7.5^ TCID_50_/mL). The plates were incubated at 37°C for 48 h. Following incubation, 10 μL of CCK8 reagent was added to each well, and the plates were incubated at 37°C for an additional 4 h. The absorbance was measured at 450 nm. The stimulation index (SI) was calculated via the following formula: SI = mean OD in test wells/mean OD in unstimulated wells.

### Detection of cytokines, ELISA antibodies, and serum biochemical parameters

Changes in serum cytokine and antibody levels were assessed via commercial ELISA kits (Meimian Industrial Co., Ltd., Jiangsu, China) for IL-12, TNF-α, IFN-γ, total IgA antibodies, total IgG antibodies, and BoAHV-1 gB antibodies. Additionally, lysozyme levels in the serum were measured via kits from Jiancheng Bioengineering Institute (Nanjing, China).

### Serum antibody detection of *M. bovis*

Serum antibodies against *M. bovis* were identified via competitive ELISA. Test serum samples, which were diluted fourfold, along with positive and negative serum controls, were combined with HRP-labeled monoclonal antibodies and added to plates coated with *M. bovis* p579 protein. The plates were incubated at 37°C for 60 min. After washing, 100 µL of substrate chromogenic solution was added, and the plates were incubated at room temperature in the dark for 10 min. The reaction was stopped, and the OD_450_ value was read immediately. The blocking rate (PI value) was calculated via the following formula: blocking rate = (1 − *S*/*N*) × 100%, where *S* represents the sample OD_450_ and *N* represents the mean OD_450_ of the negative control serum. The criteria for the test were as follows: 0.65 < OD_450_ for the negative control <2.0 and PI for the positive control >60%. A sample with a PI ≥ 41% was considered positive, whereas a PI < 41% indicated a negative result.

### Neutralization assay

Inactivated serum (heated at 56°C for 30 min) was serially diluted in a 96-well cell culture plate and incubated with 100 TCID_50_ of the BoAHV-1 HB06 virus at 37°C in a 5% CO_2_ incubator for 1 h. Following incubation, the serum-virus mixture was transferred to a new 96-well cell plate containing MDBK cells and cultured in a 5% CO_2_ incubator at 37°C for 3 days. The neutralizing antibody titers, defined as the highest serum dilutions that inhibit BoAHV-1 infection, were calculated via the Reed‒Muench method (17).

### *M. bovis* HB0801 and BoAHV-1 HB06 shedding after challenge

To quantify the shedding of *M. bovis* HB0801 post-challenge, nasal swabs were sterilized by filtration through a 0.45 μm filter and then underwent a 10-fold serial dilution. Subsequently, 100 μL of diluted filtrate was inoculated onto PPLO solid medium and incubated at 37°C under the 5% CO₂ conditions. When typical “fried egg-shaped” colonies were observed, the original concentration could be calculated based on the dilution factor.

The shedding of the BoAHV-1 HB06 viral genome after challenge was measured via extracted DNA from nasal swabs used for RT-PCR targeting the envelope glycoprotein B (*gB*) gene. The primer and probe sequences of the gB gene could be found in the previous studies (18), and the program was as follows: 95°C for 30 s, 95°C for 10 s, 60°C for 20 s, and 40 cycles of 95°C for 15 s, 60°C for 20 s, and 95°C for 15 s.

### Statistical analysis

One-way ANOVA was employed using GraphPad Prism 9 to assess significant differences between groups. The assumptions of normality and homoscedasticity were verified using the Shapiro-Wilk and Levene’s tests, respectively. These tests were chosen to ensure the accurate and reliable interpretation of differences across the experimental groups. A *P* value of less than 0.05 (*), 0.01 (**), 0.001 (***), or 0.0001 (****) was considered to indicate significant differences. The error bars represent the standard error of the mean.

## RESULTS

### Safety of EcN inoculation in dairy calves

**Fig 2:**
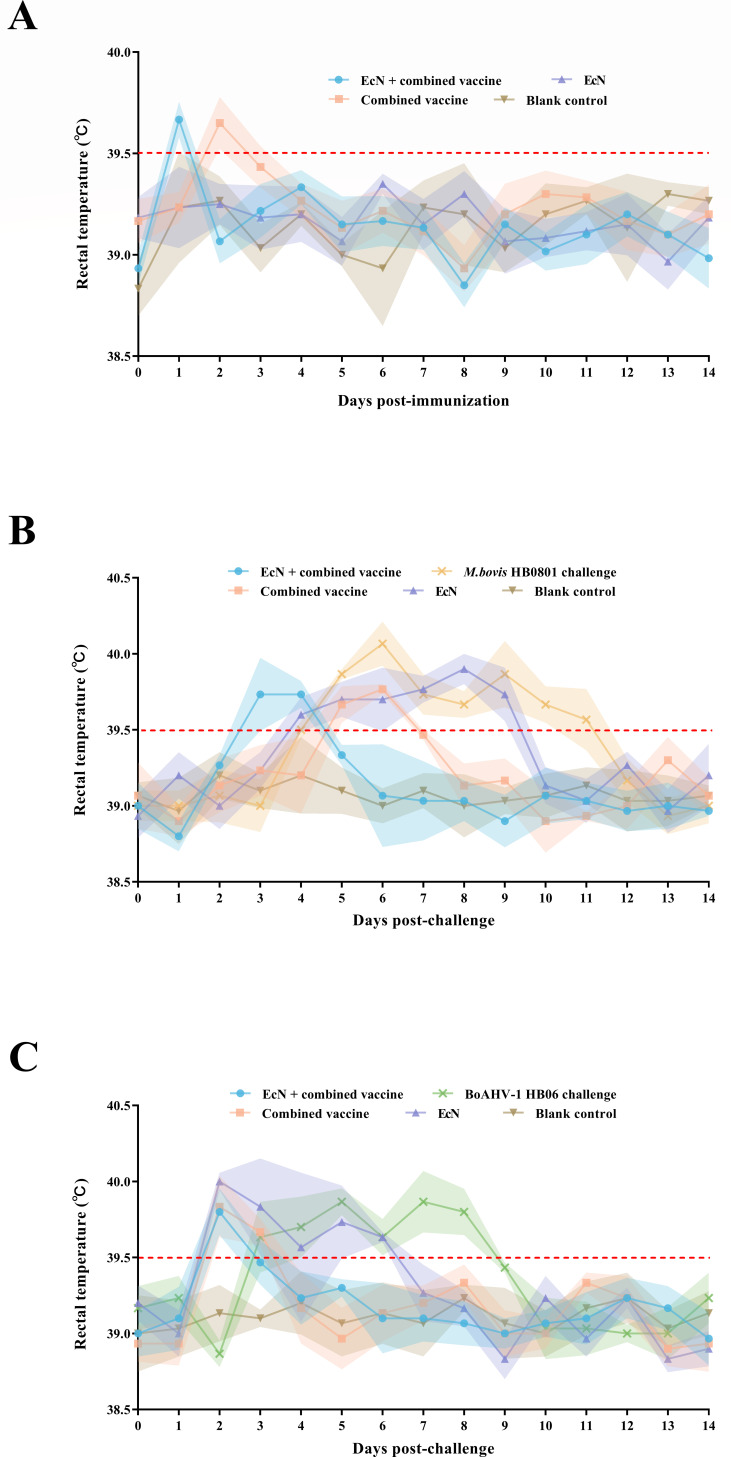
Rectal temperature changes after (**A**) inoculation and challenge with (**B**) *M. bovis* HB0801 and (**C**) BoAHV-1 HB06, respectively. The red line represents the upper threshold of normal rectal temperature.

All vaccinated calves maintained normal rectal temperatures and mental alertness, and no signs of respiratory distress were observed after EcN inoculation. Four cattle in the groups receiving the EcN + combined vaccine and the combined vaccine experienced slight increases in rectal temperature on days 1 and 2 following immunization, respectively. Besides, those cattle that received EcN alone presented no abnormalities in rectal temperature (Fig. 2A).

Following the *M. bovis* HB0801 or BoAHV-1 HB06 challenges, calves in the EcN + combined vaccine group and the combined vaccine group exhibited only a rectal temperature increase not exceeding two consecutive days. Furthermore, no clinically significant abnormalities were observed in these two groups.

After the *M. bovis* HB0801 challenge, calves in the EcN group and non-immune group exhibited rectal temperature changes on days 4–9 and 5–11, respectively, accompanied by coughing, profuse salivation, and depressed spirits. The abnormal rectal temperature persisted for 5 days in the EcN group and 6 days in the non-immune group when facing the BoAHV-1 HB06 challenge, with increased nasal and ocular discharge and the occurrence of nasal mucosal bleeding (Fig. 2B and C). These findings indicated that EcN inoculation did not adversely affect the health of dairy cattle, demonstrating its safety for enhancing vaccine immunogenicity. However, calves receiving EcN alone could not provide effective protection against challenges.

### EcN elicits innate immune responses when combined with the vaccine

**Fig 3:**
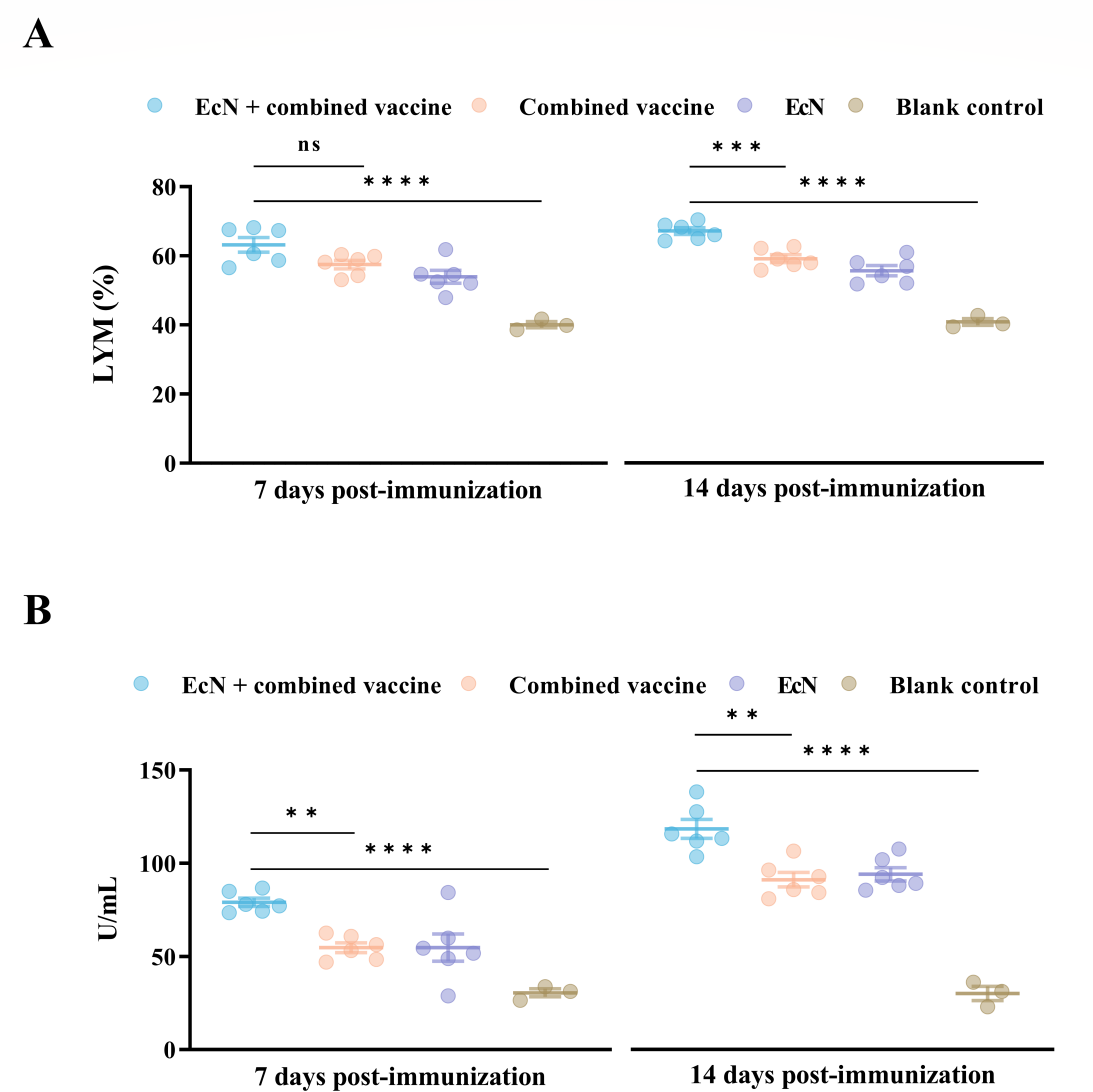
EcN enhanced the ability of the combined vaccine to stimulate innate immune responses in vaccinated calves. (**A**) The percentage of lymphocytes in whole blood and (**B**) the lysozyme content in the serum. **P < 0.01, ***P < 0.001, ****P < 0.0001; ns, no significant difference between groups.

At 7 days after immunization, the lymphocyte percentages were significantly greater in the EcN + combined vaccine group than those in the blank control group (*P* < 0.0001), but no inter-group differences were observed. By day 14, all treated groups exhibited a sustained upward trend in lymphocyte percentages, and the EcN + combined vaccine group held much higher lymphocyte percentages versus the combined vaccine group (*P* = 0.0008) and blank controls (*P* < 0.0001) at this time (Fig. 3A).

Besides, the EcN + combined vaccine group demonstrated markedly higher lysozyme concentrations than the combined vaccine group (*P* = 0.0056), which in turn had significantly higher levels than the blank control at day 7. By 14 days post-immunization, vaccinated groups showed remarkable increases in lysozyme secretion compared to 7 dpi, and the notable difference in lysozyme content between the EcN + combined vaccine group and the combined vaccine group remained (*P* = 0.001) (Fig. 3B).

Calves were able to activate corresponding innate immune responses even after receiving only EcN inoculation, achieving lymphocyte percentages comparable to the combined vaccine group and high concentrations of lysozyme.

### Lymphocyte proliferative response

**Fig 4:**
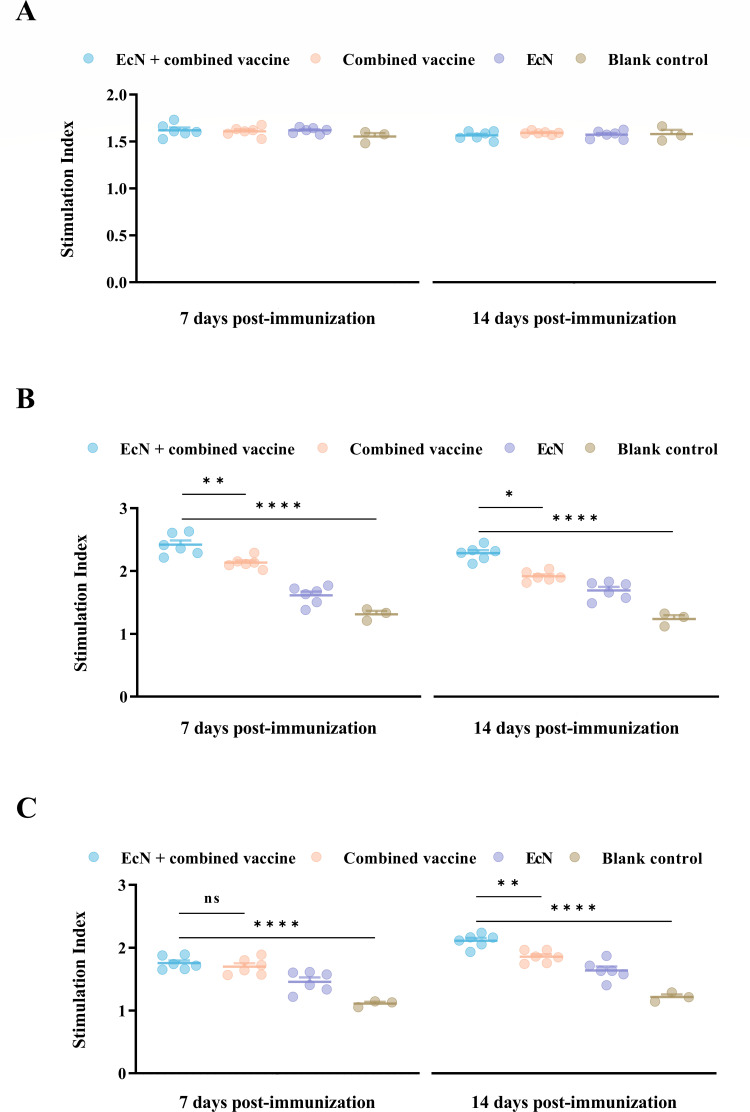
Lymphocyte proliferation assays were performed using PBMCs. (**A**) ConA served as a positive control, while (**B**) *M. bovis* protein and (**C**) inactivated BoHV-1 were used as stimulants to detect lymphocyte proliferation following inoculation. See Materials and Methods for methods. *P < 0.05, **P < 0.01, ***P < 0.001, ****P < 0.0001; ns, no significant difference between groups.

The enhancement of lymphocyte proliferation was observed in all groups (including the blank control group) at days 7 and 14 post-immunization following concanavalin A (ConA) stimulation. However, no significant differences could be found between the treated groups and the blank control group when exposed to non-specific stimuli (Fig. 4A).

The stimulation induced by the EcN-treated vaccinated group via *M. bovis* whole-cell protein was significantly greater than that of the combined vaccine group at 7 days post-immunization (*P* = 0.0089), but no statistically significant difference was shown in lymphocyte proliferation capacity between these two groups following inactivated BoAHV-1 stimulation (Fig. 4B and C).

By 14 days post-inoculation, among those *M. bovis-*treated groups, although lymphocyte proliferation capacity shifted to a decreasing trend across the treated groups, the EcN + combined vaccine group still exhibited a different result compared to the combined vaccine group (*P* = 0.034). Meanwhile, lymphocyte proliferation levels across inoculated groups displayed an opposite upward tendency at 14 dpi when stimulated with inactivated BoAHV-1, and the EcN + combined vaccine group also held superior proliferation ability compared to the combined vaccine group (*P* = 0.0075) (Fig. 4B and C).

### Activation of cellular immunity by EcN + combined vaccine

**Fig 5:**
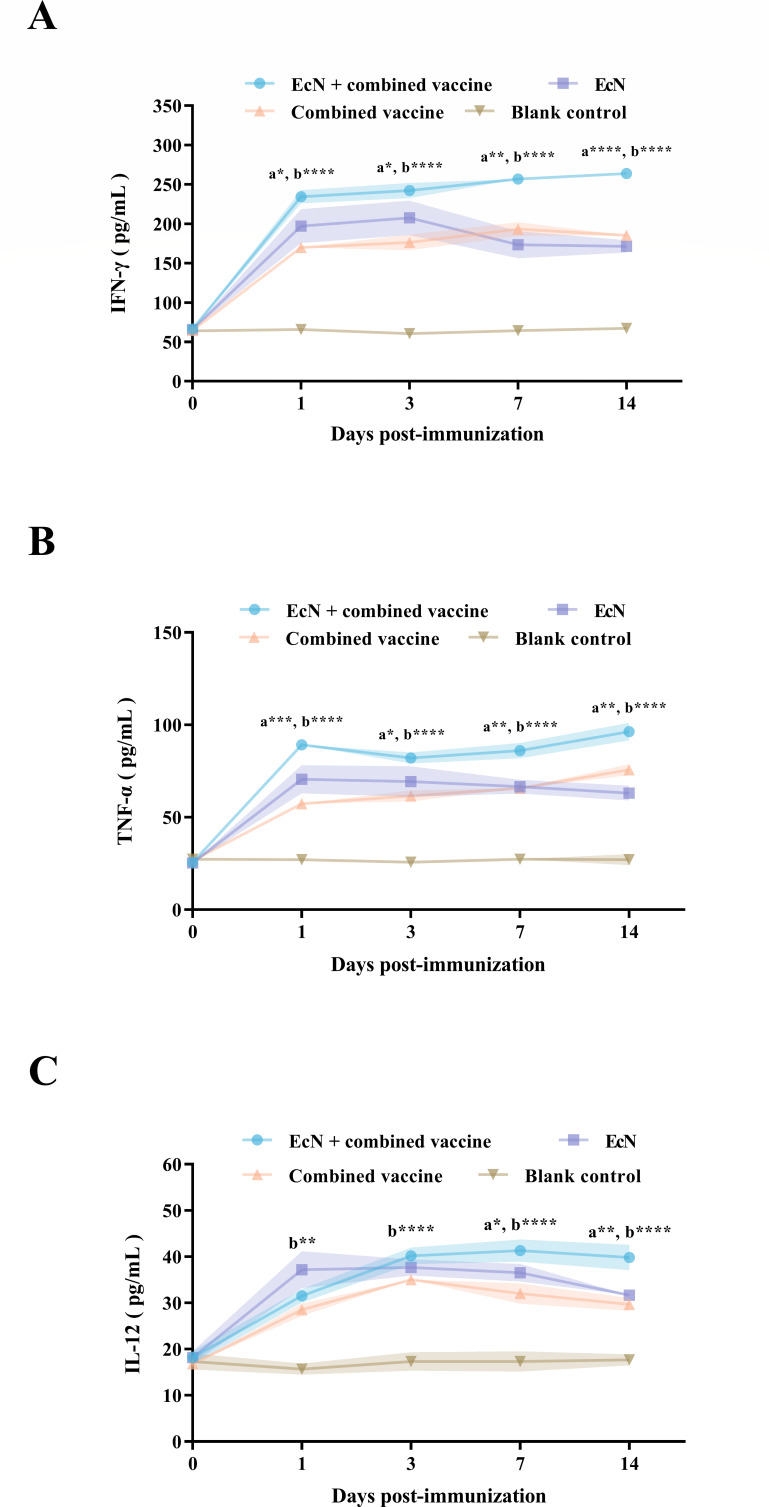
The cellular immune response elicited by the administration of the coupled with EcN and the *M. bovis*-BoHV-1 combined vaccine was illustrated. Panels **A**, **B**, and **C** represent the levels of IFN-γ, TNF-α, and IL-12, respectively. The symbols “a” and “b” represent significant differences between the EcN + combined vaccine group and the combined vaccine group, as well as between the EcN + combined vaccine group and the blank control group, respectively. The meaning of the "*" is detailed in "Statistical analysis.".

In samples collected prior to treatment (D0), all calves in experimental groups exhibited only low levels of expression for the three cytokines, maintaining baseline serum concentrations equivalent to those of the blank control calves. After inoculation, IFN-γ and TNF-α showed elevated expression levels post-treatment in the EcN + combined vaccine group, showing a steadily growing trend overall and reaching peak levels of 263.7 and 98.3 pg/mL at 14 days post-treatment, respectively. During the whole period after immunization, the expression levels of these two cytokines in this group were consistently higher than those in the combined vaccine group (*P* < 0.05 and *P* < 0.05). In contrast, the changes in IL-12 were less pronounced in the EcN + combined vaccine and combined vaccine groups and remained at nearly the same level during the first 3 days post-vaccination. The IL-12 concentration in the combined vaccine group subsequently began to decline, whereas the level in the EcN + combined vaccine group remained relatively stable. As a result, a significant difference in IL-12 levels emerged between the two groups during the observation period from days 7 to 14 (*P* < 0.05) (Fig. 5A through C). While calves administered EcN alone exhibited a rapid increase in cytokine levels, this elevation could not be maintained over time, and all three cytokines demonstrated a consistent decline throughout the observation period.

### Promoted serum-specific antibody response when administered with EcN + combined vaccine

**Fig 6:**
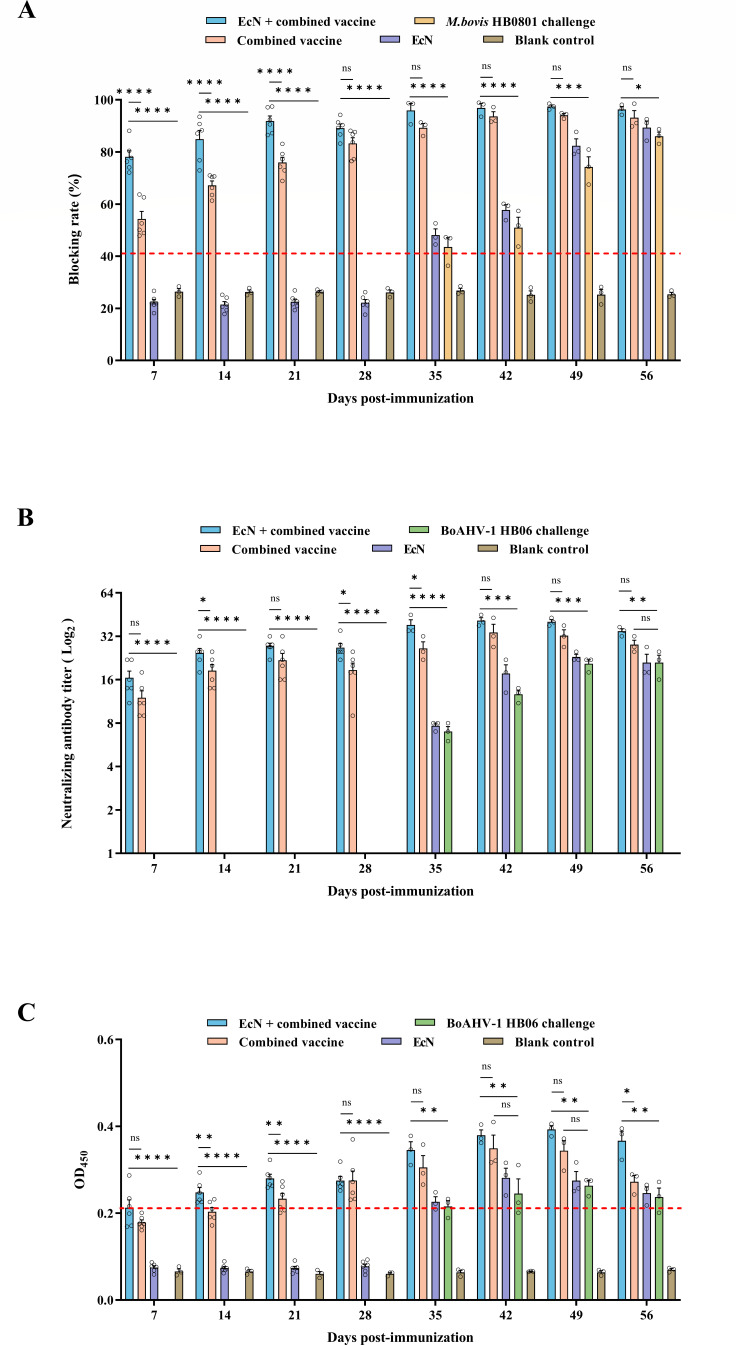
Humoral immune responses following inoculation and subsequent challenge. (**A**) Serum-specific *M. bovis* ELISA antibody, (**B**) BoAHV-1 neutralizing antibody titers, and (**C**) BoHV-1 gB antibody titers were measured. The red line denotes the negative‒positive threshold for both the *M. bovis* antibody and the gB antibody. *P < 0.05, **P < 0.01, ***P < 0.001, ****P < 0.0001; ns, no significant difference between groups.

By day 7 post-immunization, all calves in the vaccinated groups achieved seropositivity in *M. bovis* antibodies, with the EcN + combined vaccine group holding an average antibody level close to 80%, and this average antibody level reached an impressive 91.9% on day 21. Within the first 21 days after treatment, the *M. bovis* antibody levels in the EcN + combined vaccine group were significantly different compared to the combined vaccine group (*P* < 0.0001). Following the *M. bovis* HB0801 challenge, both the EcN + combined vaccine and combined vaccine groups sustained extremely high *M. bovis* antibody levels throughout, with no further obvious differences noted between the two groups (Fig. 6A).

Although both the EcN + combined vaccine and the combined vaccine groups achieved average BoAHV-1 neutralizing antibody titers exceeding the 1:8 protective threshold by day 7 post-vaccination, the EcN + combined vaccine group demonstrated a more robust antibody response and remained at a high level. This enhanced and persistent antibody response was also evident at days 14 and 28 in the post-inoculation period, where the average antibody titers differed from those of the combined vaccine group (*P* < 0.05). This inter-group difference remained observable on day 7 (35 dpi) after the BoAHV-1 HB06 challenge and vanished thereafter. But on day 28 post-challenge, the EcN + combined vaccine group still exhibited higher mean antibody titers than the non-immune group (*P* = 0.0072), a difference not found when comparing the combined vaccine and non-immune groups (Fig. 6B).

All calves in the EcN + combined vaccine group achieved seroconversion for gB antibodies at 14 days after inoculation, whereas calves in the combined vaccine group did not complete this conversion until 28 days post-vaccination. Therefore, the average gB antibody level in the EcN + combined vaccine group was notably higher than that in the combined vaccine group between 14 and 21 days post-vaccination (*P* < 0.01), and also reached its peak titer of 0.280 at 21 days post-vaccination. After the BoAHV-1 HB06 challenge, the gB antibody levels in both immune-challenge groups showed an initial increase, followed by a decrease, but the EcN + combined vaccine group was less affected, maintaining consistently higher gB antibody titers than the non-immune challenge group throughout the post-challenge period (*P* < 0.01). Meanwhile, the combined vaccine group lost its antibody advantage over the non-immune challenge group beginning at 14 days post-challenge, and at the last observation point (56 dpi), the EcN + combined vaccine group demonstrated distinct gB antibody levels compared to the combined vaccine group (*P* = 0.015) (Fig. 6C).

Meanwhile, calves treated with EcN alone could not generate pathogen-specific antibodies following inoculation.

### Enhanced IgA and IgG antibody levels when using EcN + combined vaccine

**Fig 7:**
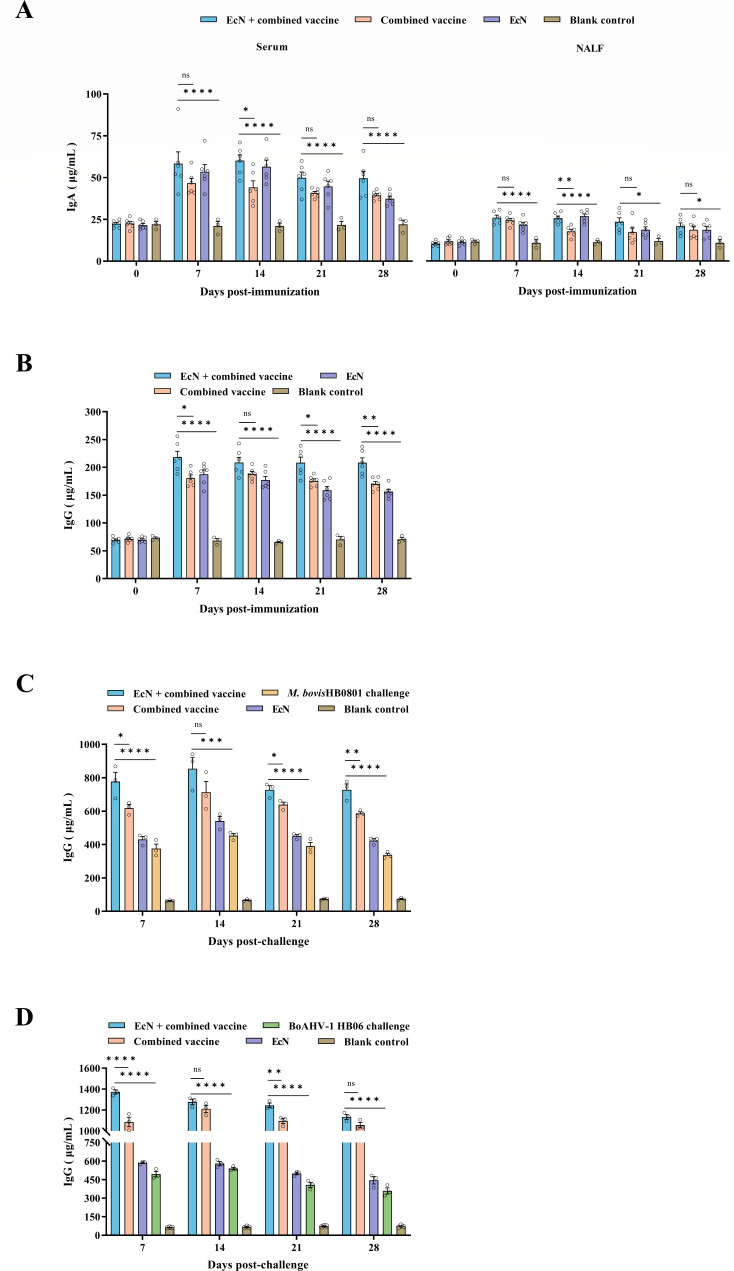
After inoculation with EcN and the *M. bovis*-BoHV-1 combined vaccine, (**A**) serum IgA levels and mucosal IgA levels in NALF, and (**B**) serum IgG antibody levels were monitored. Additionally, panels **C** and **D** illustrated the serum IgG levels after challenge with *M. bovis* HB0801 or BoAHV-1 HB06. *P < 0.05, **P < 0.01, ***P < 0.001, ****P < 0.0001; ns, no significant difference between groups.

Baseline data (D0) for all experimental groups, including the blank control group, indicated that the levels of IgA in serum and NALF samples of all calves prior to treatment, as well as the total IgG concentrations in the serum, remained at extremely low levels. Inoculation led to a moderate increase in serum IgA antibodies, with the most striking rise observed in the EcN + combined vaccine group, reaching 60.2 μg/mL by day 14, and the serum IgA antibody concentrations were also found to differ from those in the combined vaccine group at this time (*P* = 0.027). Similarly, the EcN group also showed a rapid increase in serum IgA antibody titers, with peak concentrations approaching those of the EcN + combined vaccine group. But this rapid increase did not last for a long time. In contrast, IgA levels in nasal lavage fluid remained consistently low across all experimental groups. Nonetheless, NALF IgA concentrations in the EcN + combined vaccine group were always higher than those in the blank control group (*P* < 0.05), and by day 14, its antibody levels also displayed a marked advantage over the combined vaccine group (*P* = 0.002) (Fig. 7A).

IgG antibodies were rapidly produced by cattle inoculated with the EcN + combined vaccine, presenting the greatest number of IgG antibodies at the start, peaking at 218.3 μg/mL by day 7, and remaining stable throughout the vaccination period. Hence, except for 14 dpi, the serum IgG antibody titers in this group were much higher than those in the combined vaccine group (*P* < 0.05) (Fig. 7B).

Following the challenge with *M. bovis* HB0801, the IgG antibody levels in the EcN + combined vaccine group displayed a trend of initial increase, followed by a decline, and then remained at an elevated level. At the three time points of 7, 21, and 28 days post-challenge, the serum IgG titers were obviously higher than those in the combined vaccine group (*P* < 0.05) (Fig. 7C). When the vaccinated groups were challenged with BoAHV-1 HB06, the serum IgG levels in the EcN + combined vaccine group and the combined vaccine group escalated sharply, surpassing 1,000 μg/mL throughout the post-challenge period. Among these, the EcN + combined vaccine group reached the highest titer (1,372.7 μg/mL) of all groups on day 7 post-challenge. Based on this robust secondary immune response, the IgG antibody levels in this group maintained superiority compared to the combined vaccine group at both 7 and 21 days after the challenge (*P* < 0.01) (Fig. 7D).

### EcN + combined vaccine shows shorter shedding duration and lower shedding titers after challenge

**Fig 8:**
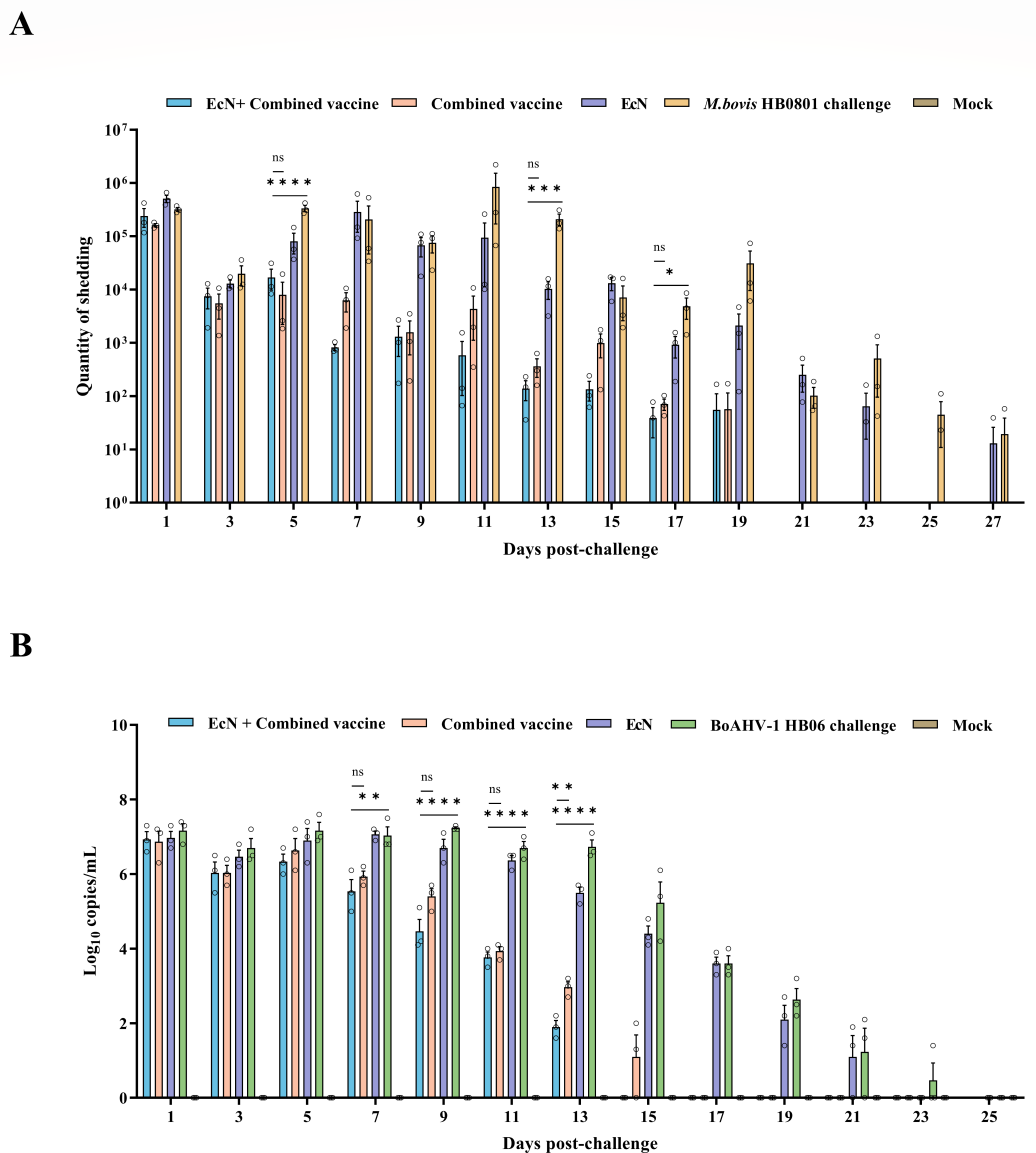
Detection of (**A**) *M. bovis* HB0801 and (**B**) BoAHV-1 HB06 shedding after challenge. *P < 0.05, **P < 0.01, ***P < 0.001, ****P < 0.0001; ns, no significant difference between groups.

When the experimental groups were challenged with *M. bovis* HB0801, all groups except the blank control group could detect high titers of *M. bovis* excretion on the first day after the challenge. Subsequently, the two immune-challenged groups (the EcN + combined vaccine group and the combined vaccine group) exhibited fluctuating but declining trends, with shedding stopped at 21 days post-challenge. Conversely, the non-immune challenge group showed elevated levels of excretion after challenge and could still be detected in the excretion until the end of the observation period of the challenge. During the after-challenge period, the non-immune challenge group demonstrated significantly higher shedding levels than the EcN + combined vaccine group at days 5, 13, and 17 (*P* < 0.05), but no statistically significant differences were observed between it and the combined vaccine groups at these time points (Fig. 8A).

After facing the challenge with BoAHV-1 HB06, virus shedding was detectable in all groups on the first day. The two immune-challenge groups showed a continuous decline starting from the fifth day onward, with the EcN + combined vaccine group consistently showing lower shedding titers than the combined vaccine group, and a noticeable difference in shedding levels between the two groups was observed by day 13 post-challenge (*P* < 0.01). By day 15 post-challenge, viral shedding disappeared in the EcN + combined vaccine group, while the combined vaccine group ceased shedding by day 17. While the non-immune challenge group maintained a high level of shedding, with a peak amount of 10^7.23^/mL on the 9th day, and maintained significantly higher shedding titers than the EcN + combined vaccine group between days 7 and 13 after the challenge (*P* = 0.0079) (Fig. 8B).

Calves exposed to EcN alone displayed lower observed and calculated shedding amounts compared to the non-immune challenge group when challenged with either *M. bovis* HB0801 or BoAHV-1 HB06. However, they failed to achieve effective protection, as neither the average shedding titers nor the duration showed superiority over the non-immune challenge group.

## DISCUSSION

BRD is a multifactorial condition characterized by complex interactions among various viral and bacterial pathogens, along with host-specific factors (19). The immune system plays a critical role in combating these infections, particularly through the interplay between innate and adaptive immunity (20–22). EcN, a special intestinal probiotic, has been extensively employed in the development of vaccines for various diseases (23–25). Recent studies have increasingly focused on the role of EcN in regulating respiratory-related diseases, capitalizing on its unique advantages as an immune booster to enhance immune responses (26). Although the results showed that EcN alone could effectively activate the innate immune response in dairy calves and induce a certain level of cellular immune response and non-specific antibody response, the primary focus of this study was not the sole application of EcN. Instead, we emphasized the promising application prospects of EcN as an immune enhancer, confirming its safety for administration to dairy calves and demonstrating its ability to markedly strengthen immune protection responses in dairy calves.

Innate immunity serves as a rapid, non-specific first line of defense against pathogens (21). Our findings demonstrated that EcN effectively activated the innate immune response in calves and enhanced the response levels induced by the vaccine. We observed increased lysozyme levels and an elevated percentage of lymphocytes in calves treated with EcN paired with the combined vaccine. This augmented immune response was markedly different from that observed in calves receiving the combined vaccine alone, indicating a robust activation of innate immune mechanisms. Lysozyme, a highly conserved antimicrobial protein, plays a crucial role in lysing bacteria, thereby contributing to the initial defense against infections (27, 28). Besides, the sustained increase in lymphocyte proliferation in the EcN-treated group further underscored the role of probiotics in promoting cellular immunity without eliciting inflammatory responses, while also suggesting a safe profile for its use in dairy calves. Meanwhile, we also acknowledge that these are non-specific parameters influenced by various physiological factors. Individually, they do not denote specific protection. However, under the strictly controlled experimental conditions, these early innate responses effectively bridge the critical gap before the onset of adaptive immunity.

In addition to successfully activating innate immune responses, the ability of EcN to stimulate and strengthen adaptive immune responses is noteworthy. Our results demonstrated that calves receiving EcN in pair with the combined vaccine presented elevated levels of key cytokines (including IFN-γ, TNF-α, and IL-12) shortly after treatment, and this elevated status persisted over time and was significantly different from that observed in calves administered EcN or the combined vaccine alone. These cytokines are crucial for orchestrating a protective immune response (29–31). Notably, IFN-γ serves as a critical marker of cell-mediated immunity, playing a pivotal role in activating T cells and enhancing the immune response to intracellular pathogens. Although *in vivo* mechanistic validations of these molecular interactions were not performed in this study, the profound immunomodulatory effects and the sustained cytokine profiles observed can be theoretically attributed to the synergistic activation of Toll-like receptor (TLR) pathways. Specifically, EcN primarily engages TLR4 and TLR5 (32, 33), while the combined vaccine components provide additional pathogen-associated molecular patterns: *M. bovis* and BoAHV-1 are recognized by TLR2/TLR6 and TLR9/TLR3, respectively (34, 35). The simultaneous binding of these diverse TLRs converges on the MyD88-dependent signaling cascade, driving NF-κB activation (34). This convergence effect triggers the robust secretion of IL-12, which acts as a main regulator to stimulate natural killer cells and naive T cells, thereby establishing a positive feedback loop for sustained IFN-γ and TNF-α production (36). Therefore, this TLR-MyD88-NF-κB-cytokine axis provides a reasonable molecular basis for the Th1-biased cellular immune response shown in this study. It clearly reveals that the use of EcN not only primes the immune system but also fundamentally enhances the efficacy of subsequent vaccination and prolongs the duration of the induced immunity.

Moreover, the findings of this study regarding the rapid production of specific antibodies further emphasized the role of EcN in enhancing humoral immunity. In comparison to the control calves, those receiving EcN combined with the vaccine showed prompt production and significantly higher titers of *M. bovis* antibodies, BoAHV-1 neutralizing antibodies, and gB antibodies. This was evidenced by a mean anti-*M*. *bovis* blocking rate of nearly 80% in the EcN + combined vaccine group by day 7. Furthermore, the EcN + combined vaccine group achieved an average BoAHV-1 neutralizing antibody titer of 1:16.5 immediately post-immunization, which significantly exceeded the protective threshold of 1:8 (37). Besides, the gB antibodies in the EcN + combined vaccine group were the first to accomplish herd seropositivity conversion by day 14 post-inoculation. Additionally, the EcN-enhanced specific antibody response maintained exceptionally high levels regardless of challenges with *M. bovis* HB0801 or BoAHV-1 HB06, demonstrating minimal decline over time. Collectively, these findings illustrated the potential of EcN as a potent immunological adjuvant. EcN significantly accelerated the generation of vaccine-specific humoral immune responses and prolonged their duration. Such a rapid and robust antibody response is crucial for providing immediate protection against pathogens (38), particularly during the vulnerable early post-vaccination period, thereby improving resistance to infections.

IgA functioned as the primary antibody in the mucosal immune system, serving as both the first chemical barrier and immune regulatory hub (39, 40), and its secretion levels also correlated positively with indicators of host pulmonary function (41). Consequently, the significance of IgA in respiratory diseases cannot be overstated (42). Our results revealed that the EcN-treated vaccinated group exhibited a substantial increase in serum IgA production, maintaining elevated levels that were remarkably higher than those observed in the combined vaccine and blank control groups. Although IgA levels in NALF did not show the expected elevation, this modest enhancement still allowed immunized calves paired with EcN to display superior IgA levels in NALF compared to the controls. Elevated IgA levels are essential for protecting mucosal surfaces against pathogen invasion, thereby contributing to overall respiratory health. Meanwhile, these findings also underscored the effectiveness of EcN in promoting vaccine-induced local immune responses, which are vital for preventing respiratory infections (43).

IgG is essential for secondary immune responses (44). Our study demonstrated that the immunized group paired with EcN showed elevated levels of IgG antibodies shortly after inoculation, with no downward trend over time. Moreover, whether challenged with *M. bovis* HB0801 or BoAHV-1 HB06, the IgG antibody response in this group surged rapidly and remained at exceptionally high levels. The rapid and effective adaptive immune responses generated by EcN are vital for controlling and eliminating pathogenic microorganisms, particularly in the upper respiratory tract, and the intensity of these immune responses also surpassed that observed in calves receiving the combined vaccine alone. This sustained IgG response is crucial for preventing diseases associated with respiratory infections, which pose major threats to cattle health. The capacity of EcN to accelerate the IgG response while enhancing its persistence fully underscores its potential as a powerful immunological adjuvant.

Monitoring of shedding amounts following the challenge with *M. bovis* HB0801 or BoAHV-1 HB06 revealed that calves administered EcN + combined vaccine or combined vaccine alone both effectively suppressed the excretion and duration compared to the non-immune challenge individuals. However, immunized calves receiving EcN displayed lower shedding titers than those receiving the combined vaccine alone when challenged with both pathogens. Although significant differences in shedding levels between the two groups emerged at only a few time points during the observation period, the shedding duration of BoAHV-1 HB06 was 2 days shorter in the EcN + combined vaccine group than in the combined vaccine group. These outcomes closely aligned with the study objectives, providing compelling evidence that co-administration of EcN with the combined vaccine further reduced shedding titers and duration, thereby highlighting EcN’s ability to elicit a robust and sustained immune response once again. These results represented a critical consideration for the practical application of EcN, as they demonstrated its efficacy in boosting vaccine-specific immune protection, providing improved protection against various pathogens, and highlighting the promising potential of EcN as a vaccine adjuvant.

### Conclusion

In conclusion, our study highlighted the significant role of EcN as an immune enhancer in dairy calves. Its capacity to activate both innate and adaptive immune responses not only improved the immunogenicity of vaccines but also facilitated immediate protection against infections under experimental challenge conditions. These compelling evidences represent the first exploratory investigation into the application of EcN in dairy calves, indicating that the incorporation of EcN into vaccination strategies could lead to improved disease resistance, particularly against BRD, and overcome the delayed immunity often associated with conventional vaccination protocols. Future research should explore broader applications of EcN in dairy cattle health, as experimental challenge conditions cannot fully replicate the complex polymicrobial and stress factors present in real-field feedlot environments. This study introduced a novel, non-antibiotic approach to enhance the immunogenicity of a combined vaccine, providing strong evidence for the utilization of EcN as a valuable tool for enhancing immune responses in veterinary medicine.

## Data Availability

The data supporting the findings of this study are available within the article. Further inquiries can be directed to the corresponding authors.
